# Clinical characteristic analysis of 25 cases of hepatic portal venous gas

**DOI:** 10.3389/fsurg.2025.1619587

**Published:** 2025-08-20

**Authors:** Changhui Ji, Lihong Zhang, Zhirong Cheng, Zhilong Jiang, Tao Ji, Bo Ye

**Affiliations:** ^1^ Department of General Surgery, Taixing People's Hospital Affiliated to Yangzhou University, Taixing, Jiangsu, China; ^2^ Department of Hepatobiliary Surgery, Affiliated Hospital of Yangzhou University, Yangzhou, Jiangsu, China; ^3^ Department of Imaging, Taixing People's Hospital Affiliated to Yangzhou University, Taixing, Jiangsu, China

**Keywords:** abdominal pain, hepatic portal venous gas (HPVG), abdominal CT, surgical intervention, prognosis

## Abstract

**Objective:**

To analyze the clinical characteristics, etiological distribution, and treatment outcomes of Hepatic Portal Venous Gas (HPVG) in a cohort of elderly patients with multiple comorbidities, and to evaluate the impact of early surgical intervention on survival rates.

**Methods:**

A retrospective study was conducted on 25 patients with HPVG admitted to Taixing People's Hospital of Yangzhou University from January 2010 to June 2024. The study included demographic characteristics, symptoms, comorbidities, etiology, laboratory and abdominal CT results, treatment, and outcomes.

**Results:**

The male-to-female ratio was 2.6:1, with a median age of 62 years. Common symptoms included abdominal pain (88%), vomiting (44%), and septic shock (36%). Comorbidities included coronary heart disease (52%), type 2 diabetes (64%), and hypertension (76%). Leukocytosis was observed in 84% of patients. Abdominal CT scans revealed HPVG in all patients. Etiologies included intestinal ischemia/necrosis (56%), intestinal obstruction (24%), suspected intestinal perforation (12%), and intestinal inflammation (8%). Treatment involved emergency surgery combined with antibiotic therapy in 72% of patients and conservative management in 28%. Outcomes showed 60% effectiveness and 40% mortality. Among the surgical group, 12 patients recovered and 6 died; among the conservative group, 3 recovered and 4 died.

**Conclusion:**

HPVG has complex etiologies, and abdominal CT is the recommended diagnostic method. Patients with acute abdomen should undergo surgery as soon as possible to improve prognosis, although some cases have poor prognosis.

## Introduction

Hepatic portal venous gas (HPVG), first described by Wolfe and Evans in 1955 in neonates with necrotizing enterocolitis (1), historically carried a grave prognosis, with Liebman et al. (1978) reporting a mortality rate of 75% (2). Advances in imaging technologies, particularly computed tomography (CT), have since reshaped its clinical understanding, revealing HPVG not as an independent disease but as a critical imaging marker of underlying abdominal pathology, including mesenteric ischemia, intestinal obstruction, iatrogenic injuries (e.g., post-endoscopic procedures), severe infections, or traumatic insults (3–6). While mortality rates have declined significantly—reaching 16.5% in intestinal ischemia cases (7)—the urgency of surgical intervention remains contentious (8). Notably, elderly patients with comorbidities such as hypertension and diabetes face heightened risks due to delayed diagnosis and therapeutic uncertainty. This study investigates the clinical profiles, etiological patterns, and treatment outcomes of HPVG in this vulnerable population, aiming to address gaps in risk stratification and optimize management strategies for improved survival.

## Patient and methods

This study complies with the Helsinki Declaration and was approved by the Ethics Committee of the Taixing People's Hospital of Yangzhou University, and the informed consent of the subjects was exempted. The approval number is 2024-TXC-038. The study was an analytical study in 25 adult patients with hepatic portal venous gas (HPVG) who were definitively diagnosed by abdominal CT examination from January 2010 to June 2024 in the Taixing People's Hospital of Yangzhou University. The medical records of these patients were summarized, including demographic data, onset symptoms, comorbidities, laboratory test results (blood routine and C-reactive protein), abdominal CT findings, treatment details, and prognosis.

Inclusion criteria were: (i) Age ≥18 years old; (ii) Definitive diagnosis of HPVG confirmed by abdominal CT examination. Exclusion criteria were: (i) Incomplete medical history at the time of onset; (ii) Unclear diagnosis or suspected intrahepatic biliary gas. Data analysis was performed using descriptive statistics to summarize patient characteristics, etiology, treatment, and outcomes.

## Results

### Clinical characteristics

Among the 25 patients, there were 18 males (72.0%) and 7 females (28.0%), with a male-to-female ratio of 2.6:1. The patients’ ages ranged from 38 to 89 years, with a median age of 62 years. Common symptoms included abdominal pain [88.0% (22/25)], vomiting [44.0% (11/25)], and septic shock [36.0% (9/25)]. The severity of abdominal pain varied, with some patients reporting severe, persistent pain requiring opioid analgesia. Additional signs included abdominal distension (*n* = 15) and peritoneal irritation (*n* = 10). Comorbidities significantly impacted patient condition, with hypertension (76%) and type 2 diabetes (64%) being the most prevalent and potentially exacerbating the severity of HPVG and its complications. (Table 1).

**Table 1:** Clinical details and outcomes of 25 patients with hepatic portal venous gas.

Case	Sex	Age	Comorbidities	Symptoms	Lab abnormalities	Etiology	Treatment	Outcome	Follow-up CT (PVG resolution)
1	M	62	HTN, T2DM, CAD	AP, V	WBC↑, N ↑, CRP↑	Intestinal ischemia	Surgery	Survived	Disappeared (3 d)
2	M	58	HTN, old cerebral	AP, SS	WBC↑, N ↑, CRP↑	Intestinal ischemia	Surgery	Death	N/A
3	F	72	HTN, T2DM, CKD	AP	N↑, CRP ↑	Intestinal obstructi	Surgery	Death	N/A
4	M	65	CAD, atrial fibrillation	AP, V, SS	WBC↑, N ↑, CRP↑	Suspected perforation	Surgery	Death	Disappeared (5 d)
5	M	48	T2DM, atherosc lerosis	AP	WBC↑, CRP↑	Intestinal ischemia	Conserv ative	Death	N/A
6	M	75	HTN, malignancy	AP, V	WBC↑, N ↑, CRP↑	Intestinal ischemia	Surgery	Survived	decreased(4 d)
7	F	89	HTN, T2DM, CAD, CKD	SS	N↑, CRP ↑	Inflamm atory bowel	Conservative	Survived	Disappeared (4 d)
8	M	55	HTN, T2DM	AP, V	WBC↑, N ↑, CRP↑	Intestinal	Surgery	Survived	Disappeared (5d)
9	M	63	CAD, old cerebral	AP, SS	WBC↑, N ↑, CRP↑	Intestinal ischemia	Surgery	Survived	Disappeared (6 d)
10	F	68	HTN, T2DM, CAD	AP	CRP↑	Intestinal obstructi	Conserv ative	Death	N/A
11	M	71	HTN, atrial fibrillation	AP, V, SS	WBC↑, N ↑, CRP↑	Intestinal ischemia	Surgery	Survived	Disappeared (3 d)
12	M	59	T2DM, CKD	AP, V	WBC↑, N ↑, CRP↑	Intestinal obstructi	Surgery	Survived	Disappeared (3 d)
13	M	66	HTN, malignancy	AP	N↑, CRP ↑	Intestinal ischemia	Surgery	Death	N/A
14	F	81	HTN, CAD	AP, SS	WBC↑, N ↑, CRP↑	Suspected perforation	Surgery	Death	N/A
15	M	53	T2DM, atherosc lerosis	AP, V	WBC↑, CRP↑	Intestinal ischemia	Conserv ative	Survived	Disappeared (3 d)
16	M	62	HTN, CAD, old cerebral	AP	WBC↑, N ↑, CRP↑	Intestinal ischemia	Surgery	Survived	Disappeared (3 d)
17	M	77	HTN, T2DM, CKD	AP, V, SS	N↑, CRP ↑	Strangulated femoral hernia	Surgery	Survived	Disappeared (3 d)
18	F	45	Malignancy	AP, V	WBC↑, N ↑, CRP↑	Intestinal obstruction	Conserv ative	Death	N/A
19	M	69	HTN, T2DM, CAD	AP	WBC↑, N ↑, CRP↑	Intestinal ischemia	Surgery	Survived	Disappeared (3 d)
20	M	38	T2DM	AP, SS	WBC↑, N ↑, CRP↑	Suspected Perforation	Surgery	Death	N/A
21	F	74	HTN, old cerebral	AP, V	N↑, CRP ↑	Intestinal ischemia	Conserv ative	Death	N/A
22	M	64	HTN, T2DM	AP, V	WBC↑, N ↑, CRP↑	Inflamm atory bowel	Conserv ative	Survived	Disappeared (5d)
23	M	57	CAD, atherosc lerosis	AP, SS	WBC↑, N ↑, CRP↑	Intestinal obstructi	Surgery	Survived	Disappeared (4 d)
24	F	83	HTN, T2DM, CKD	AP	CRP↑	Intestinal ischemia	Surgery	Survived	Disappeared (4 d)
25	M	61	HTN, atrial fibrillation	AP, V	WBC↑, N ↑, CRP↑	Intestinal obstruction	Surgery	Death	N/A

HTN, hypertension; T2DM, type 2 diabetes mellitus; CAD, coronary artery disease; CKD, chronic kidney disease; AP, abdominal pain; V, vomiting; SS, septic shock; N, neutrophils; WBC, white blood cell; CRP, C-reactive protein.

### Laboratory results

Laboratory findings showed leukocytosis (>9.5 × 109/L) in 21 patients (84.0%), increased absolute neutrophil counts (>6.3 × 109/L) in 22 (88.0%), and elevated CRP levels in 23 (92.0%).

### Abdominal CT findings

**Figure 1:**
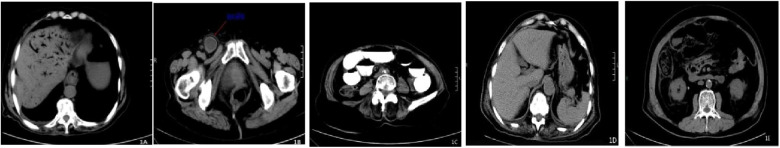
Shows the abdominal CT findings of an 89-year-old female patient with incarcerated femoral hernia and small bowel necrosis complicated by portal venous gas; **(A)** shows extensive linear gas shadows in the intrahepatic portal vein areas on cross-sectional CT, extending to under the liver capsule, presenting a typical “dead branch” dilation; **(B)** is a cross-sectional image of the incarcerated femoral hernia; **(C)** is a cross-sectional CT image showing extensive small bowel dilation and gas accumulation; **(D)** is a cross-sectional CT image taken 9 days after surgical treatment showing the disappearance of portal venous gas; **(E)** exhibited pneumatosis intestinalis, characterized by gas within the wall of the intestine.

All patients underwent abdominal CT scans, which revealed radiotransparent gas shadows in the hepatic portal venous system extending to the subcapsular space (Figure 1A). Based on medical history and CT results, the causes were analyzed as intestinal ischemia/necrosis in 14 cases (56.0%), including 1 case of incarcerated femoral hernia (Figure 1B); intestinal obstruction in 6 cases (24.0%) (Figure 1C); suspected intestinal perforation in 3 cases (12.0%); and intestinal inflammation in 2 cases (8.0%). In addition, 10 patients (40%) exhibited pneumatosis intestinalis, characterized by gas within the wall of the intestine (Figure 1E). The signs of intestinal perforation were defined as the presence of free air under the diaphragm or within the peritoneal cavity on CT scans.

### Treatment and outcomes

Among the 25 patients, 18 (72.0%) underwent emergency surgery combined with intravenous antibiotic therapy for infection control. Seven patients (38.0%) did not receive surgical treatment due to rapid disease progression, lack of surgical indication, advanced age, or multiple comorbidities, and instead received conservative treatment focused on intravenous antibiotics for infection control. Fifteen patients (60.0%) recovered well and were discharged after treatment, while 10 (40.0%) did not respond to aggressive anti-infective, anti-shock, organ function support, and fluid resuscitation therapies and died within 6 hours to 50 days of onset. Among the 18 surgically treated patients, 12 recovered and 6 died; among the 7 conservatively treated patients, 3 recovered and 4 died. Among the 14 patients with intestinal ischemia/necrosis, 11 underwent emergency surgery combined with anti-infective therapy, involving partial resection of the affected intestinal segment (with 2 cases also undergoing superior mesenteric artery embolectomy or bypass surgery). The resected intestinal segment length ranged from 15 to 200 cm, with 8 patients recovering well and being discharged, and 3 dying. One patient underwent superior mesenteric artery stenting 5 days after effective anti-infective therapy and recovered well. The remaining 2 patients received conservative medical treatment, with 1 dying and 1 recovering but later dying from another illness 50 days later. Among the 6 patients with intestinal obstruction, 4 underwent emergency surgery, and 2 received conservative medical treatment. Three patients responded positively (2 surgical, 1 conservative), while 3 died (2 surgical, 1 conservative). All 3 patients with suspected intestinal perforation underwent surgery, with 1 responding positively and the other 2 dying within 12–48 hours of onset. Both patients with intestinal inflammation recovered well after intravenous anti-infective treatment. Among the 15 patients who responded positively to treatment, abdominal CT re-examination 3–30 days after onset showed that portal venous gas disappeared in 14 cases (Figure 1D), with a median disappearance time of 3.8 days; in 1 case, portal venous gas decreased. (Table 1)

## Discussion

Hepatic portal venous gas (HPVG) is a rare imaging finding characterized by abnormal accumulation of gas within the portal vein and its intrahepatic branches (2). It can be secondary to various diseases, including gastrointestinal disorders, intestinal obstruction, mesenteric vascular diseases, anastomotic leaks, closed abdominal trauma, and liver transplantation (9, 10). Despite its association with poor prognosis, early recognition and aggressive intervention are crucial for improving patient outcomes (11).

This study summarizes 25 cases of HPVG (Hepatic Portal Venous Gas) diagnosed and treated in our hospital over a 14-year period and reveals the following findings: HPVG patients are predominantly male and elderly, often with coexisting chronic conditions such as hypertension, type 2 diabetes, and coronary heart disease. The clinical symptoms and signs of HPVG lack specificity, including abdominal pain, abdominal distension, nausea, vomiting, diarrhea, peritoneal irritation signs, and acidosis. Abdominal pain is the most common clinical manifestation, with a proportion of 88.0% in this patient group, and the most frequent underlying cause is intestinal ischemia/necrosis. Additionally, septic shock occurred in 36% of patients, with a mortality rate of 50%. The high mortality rate observed in our study (40%) underscores the critical need for prompt diagnosis and aggressive treatment of HPVG. While emergency surgery combined with antibiotic therapy was associated with a higher survival rate compared to conservative management (66.7% vs. 42.9%), the overall mortality remains high, indicating that further improvements in diagnostic and treatment strategies are needed. Future studies with larger sample sizes and multicenter collaboration are warranted to validate our findings and to identify additional prognostic factors and treatment options for HPVG.

The pathogenesis of HPVG is still unclear, but the following possible causes are currently considered. I. Increased intragastric and intestinal pressure due to various reasons leads to intestinal mucosal edema, ischemia, necrosis, and disruption of the mucosal barrier, allowing gas to enter the intestinal wall and then travel along the small intestinal veins into the mesenteric veins and back to the portal vein (12, 13). II. Gas-producing bacterial infections in the intestine result in the production of a large amount of gas that enters the intestinal wall and portal venous system (14, 15). III. Various procedure-related complications such as colonoscopy, endoscopic treatment, liver radiofrequency ablation, TIPS (transjugular intrahepatic portosystemic shunt), etc., can cause gas to enter the portal vein (16, 17). Hussain et al. (18) summarized the possible causes of HPVG as follows: intestinal ischemia and mesenteric vascular disease (61.4%), gastrointestinal inflammation (16.3%), gastrointestinal obstruction and dilatation (9.0%), sepsis (6.6%), iatrogenic injuries and trauma (3.0%), cancer (1.8%), and primary portal venous gas (1.8%). Among them, intestinal ischemia with secondary intestinal necrosis is the primary cause of HPVG. Additionally, potential causes described in literature also include intestinal perforation, intestinal fistula, acute pancreatitis, appendicitis, intra-abdominal abscesses, gastric ulcers, inflammatory bowel disease (19), diverticulitis, etc. It is important to note that HPVG often has a complex etiology, with multiple potential causes contributing to its development. In our study, we observed a diverse range of etiologies, including intestinal ischemia/necrosis, intestinal obstruction, suspected intestinal perforation, and intestinal inflammation. This heterogeneity of causes is consistent with previous studies on HPVG and underscores the need for a comprehensive differential diagnosis in patients presenting with this condition.

The diagnostic methods for HPVG include abdominal plain films, abdominal color Doppler ultrasonography, and abdominal CT scans (20–22). Abdominal plain films can only detect large amounts of portal venous gas and have low sensitivity; abdominal color Doppler ultrasonography is greatly influenced by the operator's experience and cannot diagnose the underlying cause. Therefore, both of these methods are less commonly used. Abdominal CT scans are rapid, highly sensitive, and specific, and can detect potential causes such as intestinal necrosis, intestinal obstruction, and intestinal ischemia. They are the preferred method for diagnosing HPVG. The typical CT appearance of HPVG is linear gas shadows in the portal vein territory within the liver, particularly prominent in the left lobe of the liver. Small amounts of gas accumulation may manifest as subtle linear gas shadows under the liver capsule, while large amounts of gas accumulation may result in a typical “dead branch” dilation of the portal vein. The CT manifestations of HPVG and intrahepatic biliary gas are extremely similar, and careful differentiation is required to avoid misdiagnosis due to lack of experience: Intrahepatic biliary gas is usually located in the center of the liver due to the centripetal flow of bile, typically more than 2 cm away from the liver capsule, and patients often have a history of biliary surgery or endoscopic procedures. In contrast, portal venous gas aligns with the direction of portal venous blood flow, and due to centrifugal gas flow, it can enter the peripheral zone of the liver, with a more pronounced peripheral distribution and can extend within 2 cm of the liver capsule. In this group of cases, all patients underwent abdominal CT scans for diagnosis, and enhanced CT results showed that 56% had severe stenosis, occlusion, or embolism of the superior mesenteric artery, which is consistent with mesenteric vascular disease being the most common cause reported in the literature. Since abdominal enhanced CT can detect the presence of mesenteric vascular disease, it is recommended for HPVG patients with an unclear underlying cause on abdominal CT scan to undergo abdominal enhanced CT examination.

The treatment of hepatic portal venous gas (HPVG) primarily includes medical conservative therapy and surgical intervention. There remains controversy regarding the choice between emergency surgery and conservative treatment (23). The treatment strategy mainly depends on the etiology of HPVG, which should be promptly identified to determine the need for emergency surgery (24). Abboud (9) et al. recommend emergency surgery to improve patient survival rates in cases of unknown etiology, intestinal ischemic necrosis, intestinal perforation, intestinal fistula, acute appendicitis, complex abdominal infections, peritonitis caused by abdominal trauma, etc. For other patients, conservative treatment (such as fasting, nasogastric decompression, and continuous somatostatin infusion via syringe pump, empirical anti-infective therapy and fluid replacement) can be adopted. If conservative treatment is ineffective, surgical indications should be reassessed. In addition, iatrogenic HPVG is often transient and does not require surgery. Traditionally, HPVG was considered to have an extremely high mortality rate and poor prognosis. With advancements in diagnosis and treatment, the mortality rate of HPVG has decreased to 29%–39% (12). Multivariate logistic regression analysis shows that surgical treatment is a protective factor against death in adults with HPVG; intestinal ischemic necrosis, hypertension, and tachycardia are independent risk factors for death in adults with HPVG (25). In this study, 18 patients underwent surgical treatment, and 7 opted for conservative treatment. Despite aggressive treatment, 40% (10/25) of patients still died, which is generally consistent with the mortality rate of HPVG reported in the literature. In terms of the time from onset to death, 5 patients died within 24 hours of onset, demonstrating the acute onset and rapid progression of the disease. Some patients even lost the opportunity for surgery and died. This suggests that clinicians should promptly administer potent anti-infective and supportive treatment based on the patient's condition after a definitive diagnosis of HPVG, while also seeking the etiology. Experienced surgeons should assess the need for emergency surgery to prevent delays in treatment that could lead to rapid deterioration and death. In this study, the survival rate was 66.7% (12/18) in the surgical treatment group, higher than 42.9% (3/7) in the conservative treatment group, suggesting that timely surgical treatment can effectively reduce mortality and improve prognosis for patients with HPVG who require surgery. The literature reports that the average absorption time of HPVG is 4.2 days, and the absorption time is not related to disease outcome (26). In this study, abdominal CT was reviewed in 15 patients 3–30 days after onset, and all showed disappearance or reduction of portal venous gas, with a median disappearance time of 3.8 days, which is consistent with literature reports.The median disappearance time of 3.8 days is reported as an approximate estimate, given the lack of serial CT scans. The study's reliance on single CT re-examination may underestimate the true dynamics of portal venous gas resolution. Future studies should incorporate longitudinal imaging to validate the temporal pattern.

Our study has several limitations, including its retrospective design, single-center setting, and relatively small sample size. These limitations prevent a thorough evaluation of all factors that may influence patient outcomes. Future studies with larger sample sizes and multicenter collaboration are needed to further elucidate the clinical characteristics, etiologies, and optimal management strategies for HPVG.

## Conclusions

Hepatic portal venous gas (HPVG) is a rare and critical condition in clinical practice with complex etiology. Abdominal CT scanning is the recommended diagnostic method for HPVG. Clinicians should pay sufficient attention to this condition, promptly identify and determine the underlying cause, and make decisive judgments on the need for emergency surgery, in order to reduce the mortality rate and improve the prognosis of patients with HPVG. As a single-center retrospective study, this research has limitations due to its relatively small sample size, which prevents a thorough evaluation of which patients have surgical indications and the factors that determine prognosis. Therefore, there are still deficiencies in this study. In the future, it is necessary to further expand the sample size or conduct multicenter studies to more fully investigate the characteristics of HPVG.

## Data Availability

The raw data supporting the conclusions of this article will be made available by the authors, without undue reservation.
